# A critical re-evaluation of the regression model specification in the US D1 EQ-5D value function

**DOI:** 10.1186/1478-7954-10-2

**Published:** 2012-01-13

**Authors:** Kim Rand-Hendriksen, Liv A Augestad, Fredrik A Dahl

**Affiliations:** 1 Health Services Research Centre, Akershus University Hospital, Lørenskog, Norway; 2 Institute of Psychology, University of Oslo, Oslo, Norway

**Keywords:** EQ-5D, tariff, regression model, misspecification

## Abstract

**Background:**

The EQ-5D is a generic health-related quality of life instrument (five dimensions with three levels, 243 health states), used extensively in cost-utility/cost-effectiveness analyses. EQ-5D health states are assigned values on a scale anchored in perfect health (1) and death (0).

The dominant procedure for defining values for EQ-5D health states involves regression modeling. These regression models have typically included a constant term, interpreted as the utility loss associated with any movement away from perfect health. The authors of the United States EQ-5D valuation study replaced this constant with a variable, D1, which corresponds to the number of impaired dimensions beyond the first. The aim of this study was to illustrate how the use of the D1 variable in place of a constant is problematic.

**Methods:**

We compared the original D1 regression model with a mathematically equivalent model with a constant term. Comparisons included implications for the magnitude and statistical significance of the coefficients, multicollinearity (variance inflation factors, or VIFs), number of calculation steps needed to determine tariff values, and consequences for tariff interpretation.

**Results:**

Using the D1 variable in place of a constant shifted all dummy variable coefficients away from zero by the value of the constant, greatly increased the multicollinearity of the model (maximum VIF of 113.2 vs. 21.2), and increased the mean number of calculation steps required to determine health state values.

**Discussion:**

Using the D1 variable in place of a constant constitutes an unnecessary complication of the model, obscures the fact that at least two of the main effect dummy variables are statistically nonsignificant, and complicates and biases interpretation of the tariff algorithm.

## Background

The EQ-5D, a generic instrument for measuring health-related quality of life (HRQoL), is used extensively in cost-utility/cost-effectiveness analyses [1,2]. The EQ-5D measures health along five dimensions (mobility, self-care, usual activities, pain/discomfort, and anxiety/depression). Each of these dimensions can be described at three levels of functioning, corresponding to (1) no problems, (2) some problems, and (3) extreme problems. This gives a total of 243 possible combinations, or health states. Specific health states are often referred to using a five-digit number, corresponding to the level of functioning for the five dimensions in the previously presented order. Thus, 11111 refers to the best state, and 33333 refers to the worst. To allow calculations and comparisons involving different impairments of health, all EQ-5D health states are assigned values using a common metric, usually such that perfect health has a defined value of 1, and death has a defined value of 0. Since EQ-5D health states (with the exception of state 11111) do not have intrinsic values on this common scale, such *tariffs *of values have usually been set through national valuation studies that ask the general population to value EQ-5D health states in relation to perfect health and death [3].

The United States EQ-5D valuation study from 2003 [4] brought improvements to EQ-5D valuation methodology by bringing in more complex sampling and population weighting schemes to achieve population representativeness and has partially superseded its 1993 British predecessor [5] as the EQ-5D valuation study of reference. Several papers have been published discussing differences and similarities between the US and United Kingdom valuation studies [6-8]. Since directly valuing all EQ-5D health states was considered impractical, EQ-5D valuation studies have typically elicited values for a subset of the EQ-5D health states, and values for the full set have been assigned using regression modeling, making the regression procedure and model crucial components of the EQ-5D system [5].

The authors of the US valuation study [4] performed numerous advanced statistical analyses to determine the best regression method and specification to predict US tariff values for the EQ-5D system. The chosen regression model differed from the preceding British model [5] in several ways, the most important of which were the lack of a constant signifying any movement away from perfect health and the inclusion of an ordinal value "D1," signifying the number of movements away from perfect health, beyond the first. The authors argued that replacing the constant with the D1 variable improved the model because "... In previous valuation studies, the constant term has been interpreted as a measure of any movement away from perfect health (i.e., a level 2 or 3 in any dimension). Including a constant term, however, yields a predicted value of < 1.0 for full health and complicates estimation of the marginal effects for the dummy variables that represent the EQ-5D descriptive system. [...] Its [the D1 variable's] use in place of a constant yielded a predicted value of 1.0 for full health, had no impact on the predicted values for other states, and allowed us to estimate directly the marginal effects of the main effect dummy variables..."[4] (p. 208, fourth paragraph, brackets inserted).

The EuroQol group has recently released official versions of the new EQ-5D-5L [9], where each dimension can be described at five levels, as opposed to the previous three. It is likely that a number of new national tariffs will be published over the next couple of years, both for countries for which EQ-5D-3L tariffs exist, and for countries without any EQ-5D tariffs.

The aim of this study was to illustrate how the use of the D1 variable in place of a constant is problematic, because it constitutes an unnecessary complication of the regression model, obscures the fact that three of the main effect dummy variables are statistically nonsignificant, and complicates and biases interpretation of the tariff algorithm.

## Methods

### Data

Analyses were performed directly on the published D1 regression model, the D1 tariff algorithm, and the predicted tariff values for all 243 health states [4].

### The D1 variable, model, and tariff values

In the literature, "D1" refers to the D1 variable, the regression model using the D1 variable, and the tariff algorithm based on the regression model. To disambiguate this, we will refer to the original D1 regression model as D1o ("o" for original) and specify whether we are referring to variables in the model or to their respective coefficients. D1 will refer to the variable in the model. The D1o regression model consists of 10 dummies (M2, S2, U2, P2, A2, M3, S3, U3, P3, and A3) corresponding to the five dimensions at level 2 (moderate problems) and 3 (extreme problems). The variables I2 and I3 correspond to the number of dimensions at levels 2 and 3, beyond the first; the squares of I2 and I3 are called I22 and I32; and the D1 variable signifies the number of dimensions beyond the first, not at level 1. The variable I2 was statistically nonsignificant in the calculation of tariff values and was excluded from the final modeling. Model calculations were performed on disutilities; 1 - time trade-off (TTO) values were such that 0 corresponded to health state 11111 (all five dimensions at level 1), and 1 corresponded to the defined value of death. Column one of Table 1 lists the published US D1 tariff and standard errors (SE) for the coefficients. Using this tariff algorithm, values for each health state can be calculated as 1, the value of perfect health, minus the predicted disutility of the health state.

**Table 1:** D1 model recalculation, tariff, and variance inflation factors (VIFs)

	D1o			D1c	
			
Parameter	Coeff. (SE)	VIF	Recalculation	Coeff. (SE)	VIF
Constant	N/A	N/A	-D1	-.140 (.010)	N/A
D1	.140 (.010)	113.184	D1-D1	N/A	N/A
					
M2	-.146 (.008)	5.336	M2+D1	*-.006 (.006)	1.913
S2	-.175 (.008)	5.336	S2+D1	-.035 (.007)	1.913
U2	-.140 (.008)	5.336	U2+D1	*-.000 (.006)	1.913
P2	-.173 (.008)	5.336	P2+D1	-.032 (.007)	1.913
A2	-.156 (.008)	5.336	A2+D1	-.016 (.007)	1.913
					
M3	-.558 (.016)	5.038	M3+D1	-.418 (.012)	3.591
S3	-.471 (.016)	5.038	S3+D1	-.331 (.013)	3.591
U3	-.374 (.013)	5.038	U3+D1	-.234 (.010)	3.591
P3	-.537 (.020)	5.038	P3+D1	-.397 (.015)	3.591
A3	-.450 (.015)	5.038	A3+D1	-.310 (.012)	3.591
					
I3	.122 (.018)	38.529	I3	.122 (.014)	21.211
I2 squared	-.011 (.002)	5.384	I22	-.011 (.002)	3.893
I3 squared	.015 (.003)	9.773	I32	.015 (.003)	7.066
					

* Statistically nonsignificant (p < .05).

### Handling the constant and state 11111

In the TTO method used for valuing health states in the US valuation study, "death" and EQ-5D state 11111 (perfect health) function as anchors for valuing the other health states. In order to be in line with the quality-adjusted life year (QALY) model [10], health state 11111 was given a fixed value of 1, and death was given a fixed value of 0. Since regression was performed using disutilities, and only impaired health states were valued, the intercept term (if allowed) represented an estimate of the common utility loss associated with all measured health states. Interpreted as a constant, this intercept clearly does not apply to state 11111. Therefore, state 11111 should be handled in one of two mutually exclusive ways. It could be considered either as outside the scope of the prediction algorithm, since it is given the axiomatic value of 1. In this case, the intercept should be interpreted as a constant applying to all health states within the prediction scope. Or state 11111 could be included in the scope of prediction, in which case the intercept should be interpreted as a nonconstant parameter applying to all health states except 11111. In either case, the predicted value for state 11111 would be 1. For the statement "Including a constant, however, yields predicted value of < 1.0 for full health" to hold true, the state must be considered inside the scope of prediction, while the intercept is interpreted as a constant.

In this paper, we considered the given value of state 11111 to be axiomatic, and therefore, outside of the scope of the tariff prediction. We interpreted the intercept as a constant within the scope of the 242 remaining EQ-5D health states.

### Analysis

#### D1c and statistical significance

We formulated the mathematical equivalent of the D1o model, including a constant and excluding the D1 variable, hereafter referred to as the D1c model ("c" for constant). Using the D1c regression model on the US valuation data, we illustrate how the choice of model influences the apparent statistical significance of the different dummy variable coefficients.

#### Multicollinearity

The D1o and D1c models are mathematically identical in terms of explained population variance and predicted values for all EQ-5D health states. However, different predictors result in different degrees of multicollinearity, a measure of the correlation between (and thereby, redundancy of) the predictors in the model. Multicollinearity is usually measured in terms of variable inflation factors (VIFs) for their predictors [11]. VIFs are readily available as optional output when performing regression modeling in most statistical software packages (SPSS/PASW, STATA, SAS, R, etc.). However, direct calculation of VIFs is also relatively straightforward, and can be done in two steps.

To calculate the VIF for any specific predictor variable in a regression model, the first step is to run a regression with the predictor in question as the dependent variable and all other predictors as independent variables. From this regression, we are only interested in the coefficient of determination (the R^2 ^term). The VIF equals 1/(1-R^2^). The square root of the VIF is a measure of how much larger the standard error term for the variable in question is as compared to the size it would have if the model was specified in such a way that the variable was uncorrelated with the other variables in the model.

Ideally, VIF values should be low, and while rules of thumb should be used with caution, cut-offs of 5, 10, and sometimes 30 have been suggested as indicating problematic levels of multicollinearity [11]. The VIFs are determined entirely by the correlation between the predictors. We calculated each VIF score of the D1o and D1c models within the definition space of the 242 EQ-5D health states.

#### Simplicity of interpretation

For the purpose of comparison, we divided the 242 EQ-5D health states into three groups: the 10 health states describing impairment in only one dimension, the 40 health states with impairments in two dimensions, and the 192 states with impairments in three or more dimensions. We considered the number of calculation steps necessary for the three groups of health states based on the two tariff models (D1o and D1c) and the implications for interpretation. The number of calculation steps necessary is relatively unimportant in most applications, but we include these analyses because the authors of the US valuation study argue for the simplicity of calculation in their paper. We also compared the calculation steps based on the D1o and D1c models for a set of example EQ-5D health states to enable a discussion of the appropriate interpretation of the parameters.

## Results

Tariff coefficients from the D1o and D1c models shared a simple mathematical relationship: I3, I22, and I32 were identical and the constant in the D1c model was the negative opposite of the D1 model coefficient. Using the D1 variable in place of a constant entails adding the value of the constant to each of the 10 dummy variable coefficients. These are not empirical results, but mathematical/logical properties of the two models; we used empirical tests merely to rule out logic errors. The published D1o tariff, its D1c counterpart, and the calculation steps between the two are listed in Table 1. The D1o and D1c tariffs resulted in identical values for all 242 predicted EQ-5D health states. The calculation steps for four example health states (11111, 22222, 33133, and 33233) are shown in Table 2.

**Table 2:** Calculation examples for the D1o and D1c tariffs

	**Model**		**Health states**											
	
			**11111**			**22222**			**33133**			**33233**		
	**D1o**	**D1c**	*	**D1o**	**D1c**	*	**D1o**	**D1c**	*	**D1o**	**D1c**	*	**D1o**	**D1c**
									
Perfect health	1	1		1	1		1	1		1	1		1	1
														
Const.	N/A	-.140	*0*			*1*		-.140	*1*		-.140	*1*		-.140
														
M2	-.146	-.006	*0*			*1*	-.146	-.006	*0*			*0*		
S2	-.175	-.035	*0*			*1*	-.175	-.035	*0*			*0*		
U2	-.140	-.000	*0*			*1*	-.140	-.000	*0*			*1*	-.140	0
P2	-.173	-.032	*0*			*1*	-.173	-.032	*0*			*0*		
A2	-.156	-.016	*0*			*1*	-.156	-.016	*0*			*0*		
														
M3	-.558	-.418	*0*			*0*			*1*	-.558	-.418	*1*	-.558	-.418
S3	-.471	-.331	*0*			*0*			*1*	-.471	-.331	*1*	-.471	-.331
U3	-.374	-.234	*0*			*0*			*0*	0	0	*0*	0	0
P3	-.537	-.397	*0*			*0*			*1*	-.537	-.397	*1*	-.537	-.397
A3	-.450	-.310	*0*			*0*			*1*	-.450	-.310	*1*	-.450	-.310
														
I3	.122	.122	*0*			*0*			*3*	.366	.366	*3*	.366	.366
I22	-.011	-.011	*0*			*16*	-.176	-.176	*0*			*0*		
I32	.015	.015	*0*			*0*			*9*	.135	.135	*9*	.135	.135
														
D1	.140	N/A	*0*			*4*	.560		*3*	.420		*4*	.560	
														
Sum				1	1		.594	.594		-.100	-.100		-.100	-.100

* Multiplier.

The D1o model displayed greater VIFs than the D1c model for all predictors (see Table 1). The highest VIFs were greater than the suggested cut-off values in both models. In the D1c model, the I3 variable had the highest VIF value, at 21.2. In the D1o model, the I3 VIF increased to 38.5, but the D1 variable VIF was substantially higher, at 113.2.

Since multicollinearity inflates the standard errors, and the D1o model suffers from higher levels of multicollinearity than the D1c model, the standard errors for the D1c model are smaller than the ones for the D1o. Using the D1c specification, two of the level 2 dummies (M2 and U2) were consistently statistically nonsignificant (p < .05), while the remaining three were close to zero.

For the 10 health states describing impairments only in a single dimension, calculation of utility values was simpler when using the D1o model than when using the D1c model; for these states, the D1 coefficient was not invoked, meaning that the D1c model required one calculation more than the D1o model. For the 40 health states with two impaired dimensions, the two models required the same number of calculations; the D1 variable equaled the negative opposite of the D1c constant. For the remaining 192 health states with three or more impaired dimensions, the D1o model required more calculation steps than the D1c, since the D1 coefficient had to be multiplied by the D1 variable, an operation that is not required for the D1c constant. While the D1o model uses perfect health as the starting point of health state values (1 - disutility), the calculations of D1c health state values can be simplified by using "any impaired health state" as the starting point ((1 - constant) -disutility, or .860 - disutility). If the latter alternative was chosen, the 10 health states with impairment in one dimension required the same number of calculation steps in each model, while the remaining 232 health states required fewer calculation steps using the D1c model.

## Discussion

The D1o and D1c models are mathematically equivalent and the two models display a simple mathematical relationship: the constant and the D1 variables are equal in magnitude, and the dummies are shifted away from zero by the value of the constant when it is replaced with the D1 variable. Using the D1c specification, two of the level 2 dummies are statistically nonsignificant.

Shifting all the dummy variable coefficients away from zero by the value of the constant makes them pass the test of statistical significance. Adding a regression variable that shifts other parameter estimates away from zero, thereby making them statistically significant, seems questionable, at least when the additional complexity of the model is not accompanied by other advantages. We are not generally opposed to including nonsignificant parameters in such regression models, but we must point out that the authors of the D1 tariff excluded the I22 variable because it was statistically nonsignificant. If the D1c specification is considered more appropriate, the I22 variable should be included or the nonsignificant dummy variables should be excluded. Removing the nonsignificant dummies would simplify the model considerably, while the resulting health state values would remain virtually unchanged.

The VIF scores for all D1o predictors were larger than those for their D1c counterparts. With suggested cut-offs of somewhere between five and 10, all the predictors display questionable levels of multicollinearity using the D1o model, while the I2 and I3 variables are questionable in both models. In addition to increasing the VIF of all other variables, the D1 variable itself had a VIF of more than 113, which is extreme in relation to the suggested cut-offs. While the reported SE estimates for the D1c coefficients aren't greatly reduced as compared to the D1o counterparts, the use of the D1o specification increases the SE of the estimated health state values, particularly for states with impairments on multiple dimensions. Consider when all five dimensions are impaired that the D1 variable is 4. This means that the SE of the D1 term is invoked four times, while the SE of the constant (in the D1c model) is invoked only once. Thus, using the D1c specification reduces the uncertainty around health state estimates.

With the exception of the very mildest health states, the D1c model required fewer calculation steps than the D1o model. Use of the D1 variable also implies more complex assumptions than suggested by the use of a constant; the use of a constant implies that the observed gap between perfect health and all other tariff values reflects a general tendency for substantial reductions in preference for all imperfect health states. The use of the D1 variable, on the other hand, implies that respondents attribute specific, additive losses in quality of life to specific impairments of health, but that they also include large "disutility discounts" to all health states where more than one dimension of health is impaired. Consider the calculations for health state 22222 (see Table 2); compared to the D1c calculations, the process of adding the constant five times (one time for each of the five dummy coefficients), then retracting it four times (the D1 variable) inflates the apparent magnitude of the dummy coefficients, while hampering interpretation. The D1o model has, as the authors of the US valuation study paper point out, a minor advantage in that it simplifies the calculation of marginal utility loss associated with single-dimension impairment. Unfortunately, the inflated dummy variable coefficients obscure two important properties of the tariff: that the utility loss of any movement away from perfect health is disproportionally large as compared to similar movements given other problems, and that the general public barely distinguishes between the five dimensions when they are at level 2 (moderate problems).

When considering the tariff algorithm, the relative magnitude of the different parameters may often be interpreted as a measure of their relative importance. Thus, the use of the D1o specification leads to the erroneous impression that level 2 problems are somewhere between one half and one quarter as serious as level 3 problems. However, the inflated dummy variable coefficients in the D1o model may systematically bias how people perceive the tariff when presented with the calculation algorithm. From the D1c model, it is apparent that adding level 2 problems to any reduced health state is associated with very little additional utility loss, except when invoking I2/I22. For "usual activities," the estimated utility loss of movement from level 1 to level 2 is zero. This has the interesting effect that, for instance, health states 33133 and 33233 have the same tariff values (see calculation examples in Table 2). This applies to all pairs, 15 in total, of states where "usual activities" moves from 1 to 2, while no other dimension is at level 2 (the 16^th ^such pair, 11111 and 11211, is associated with a utility loss of .140, the value of the constant).

We contend that there are numerous problems with replacing the constant signifying any movement away from perfect health with the D1 variable. First, it constitutes a breach of the scientific guiding law of parsimony (often referred to as Ockham's razor), in that it introduces new premises that unnecessarily complicate the model without adding to its explanatory value. Second, the substitution complicates, and potentially biases, interpretation of the tariff. Third, it obscures the fact that the dummies representing moderate problems with mobility, usual activities, and anxiety/depression are not significantly different from zero, while the two remaining level 2 dummy coefficients are both close to zero. Finally, it obscures the disproportionate utility loss of any movement away from perfect health as compared to similar movements from reduced health to further reduced health.

In this paper, we have focused on the problems related to the way the authors of the US valuation study replaced the constant term from previous regression models with the D1 variable. However, this is not the only alteration they made to the regression model used in the UK valuation study, in which the authors used a model consisting of the 10-dimension dummy variables present in the D1o model, a constant term, and dummy N3, representing the presence of any level 3 problems. In the D1o model, the N3 was replaced by the I3, representing the number of dimensions at level 3, beyond the first. Substituting the N3 with the I3 has an effect similar to the introduction of the D1 variable, in that it inflates the values of the level 3 coefficients. Replacing the I3 variable in the D1o model with the N3 dummy variable results in identical predicted values for all EQ-5D health states, while further reducing the multicollinearity of the model. The VIF for N3 would be 3.01 when substituting the I3 in the D1c model, while the VIFs of the other variables would remain unchanged. Nevertheless, substituting the N3 with I3 is less problematic than replacing the constant term with the D1 variable, since this procedure does not alter the statistical significance of any of the coefficients.

## Conclusion

The US EQ-5D tariff would be better represented using the D1c notification presented in this paper. While this would not alter any of the tariff values for the EQ-5D health states, it would reduce the redundancy of the tariff algorithm, increase its interpretability, and make the problems with the tariff (the disproportionate drop from perfect health to any impaired state and the lack of distinction between level 2 impairments) more apparent. We recommend that researchers using regression modeling to calculate EQ-5D tariffs in the future avoid using variables like the D1.

## List of abbreviations

EQ-5D: EuroQol five-dimension inventory; HRQoL measure developed by the EuroQol group. HRQoL: Health-related quality of life. TTO: Time trade-off; method for valuing health states in relation to perfect health and death. VIF: Variance inflation factor; measure of redundancy in regression models

## Competing interests

The authors declare that they have no competing interests.

## Authors' contributions

KRH and LAA conceived of the study. KRH performed all analyses and drafted the manuscript. LAA and FAD participated in the design of the study and helped draft the manuscript. All authors read and approved the final manuscript.
